# Organic Farming as an Alternative Maintenance Strategy in the Opinion of Farmers from Natura 2000 Areas

**DOI:** 10.3390/ijerph19073793

**Published:** 2022-03-23

**Authors:** Adam Pawlewicz, Wojciech Gotkiewicz, Katarzyna Brodzińska, Katarzyna Pawlewicz, Bartosz Mickiewicz, Paweł Kluczek

**Affiliations:** 1 Department of Agrotechnology and Agribusiness, Faculty of Agriculture and Forestry, University of Warmia and Mazury in Olsztyn, ul. Oczapowskiego 8, 10-719 Olsztyn, Poland; wgot@uwm.edu.pl (W.G.); katarzyna.brodzinska@uwm.edu.pl (K.B.); 2 Department of Socio-Economic Geography, Faculty of Geoengineering, University of Warmia and Mazury in Olsztyn, Prawocheńskiego 15, 10-720 Olsztyn, Poland; katarzyna.pawlewicz@uwm.edu.pl; 3 Department of Regional and European Studies, West Pomeranian University of Technology, 70-310 Szczecin, Poland; bartosz.mickiewicz@zut.edu.pl; 4 Interdisciplinary Humanistic Doctoral Studies, Faculty of Applied Linguistics, University of Warsaw, Szturmowa 4, 02-678 Warsaw, Poland; pawel.kluczek@student.uw.edu.pl

**Keywords:** organic farming, conventional farming, Natura 2000, protected areas, livelihood, farm’s economic situation

## Abstract

Agricultural land accounts for approximately 40% of the total Natura 2000 (N2K) network area. Therefore, many habitats and species protected under the Habitats and Birds Directives are dependent on or linked to agricultural practices. This implies that sustaining agriculture of a high natural value is a priority in achieving the aim of halting the loss of biodiversity in the European Union (EU). However, extensive agriculture is unprofitable in many regions of the EU, which results in it being either abandoned or intensified in the absence of financial support. Hence, organic farming (OF), which is most often supported with public funds, can be an alternative to conventional agriculture in N2K areas. This article is an empirical study of the differences in perceiving the possibilities of farm functioning in a protected area (PA) by organic and conventional farm owners. It was examined whether this could be the actual path to improving farmers’ living conditions in the context of legal protection of naturally valuable areas. The study material comprises the results of a survey conducted at the turn of 2016 and 2017, which addressed a total of 292 farmers, including 152 organic farm operators and 140 conventional farm operators, whose areas under cultivation were located within the N2K “Dolina Biebrzy” (“Biebrza Valley”) PLH200008 area in Poland. For the analysis of the data collected using structured questionnaires, a variety of statistical methods and techniques were applied. The study results indicated that in terms of satisfaction with the economic performance of their farms, there is no major difference between the opinions expressed by organic and conventional farm owners. However, organic farming could be an alternative livelihood strategy from the environmental policy perspective.

## 1. Introduction

The modern approach to environmental protection is based on the concept of caring for valuable components of both animated and inanimate nature. Protected areas (PAs) are the most widely used tool of biodiversity conservation policy [1,2,3] that are becoming increasingly important, not only for the need to protect biodiversity but also because they are closely linked to the adaptation to climate change [4,5,6].

In Europe, the pillar of nature conservation is the Natura 2000 (N2K) network, an ecological network of areas established to protect habitats and species of Europe-wide importance [7]. The natural environment in the European Union (EU) is at risk due to the large population and a high living standard, resulting in exceptionally great economic and social pressure on the environment. Not only are the progressive urbanisation and the development of infrastructure robbing the nature of more and more areas, but they are also irretrievably destroying them [8]. In order to counteract these hazardous processes, the EU took a number of measures to protect the natural environment as early as the 1970s. Therefore, the purpose of the established N2K area network is to protect biodiversity without excluding human activities, provided that they do not pose a hazard to species and habitats of conservation interest [9,10]. The essential idea is based on the approach which fully recognises that humans are an integral part of nature and that these two components must collaborate [11].

N2K is one of the world’s largest conservation networks, which currently covers over 18% of the land territory and over 8% of the maritime territory of the EU [12,13]. On the other hand, rural regions account for 88.2% of the EU territory (these are mostly rural or intermediate areas) while containing the vast majority of its natural resources [14]. Agricultural land accounts for approximately 40% of the total N2K network area. Therefore, many habitats and species protected under the Habitats [9] and Birds [15] Directives are dependent on or linked to agricultural practices. The survival of many habitats and species is dependent on extensive local farming. As a high biodiversity level usually coincides with low agricultural productivity, most of the agricultural land in the N2K network is located in agricultural areas that are less significant in economic terms [11]. It is worth stressing that traditional agricultural landscapes provide, in a natural manner, a number of ecosystem services, e.g., soil fertility, water and climate regulation, and aesthetic and cultural benefits [16,17,18,19,20]. This implies that sustainable agriculture of a high natural value (HNV) is a priority in achieving the aim of halting the loss of biodiversity in the EU. However, extensive agriculture is unprofitable in many regions of the EU, which results in it being either abandoned or intensified in the absence of financial support [11,21,22]. New solutions are needed to make this production system more profitable while sustainably increasing food production and the conservation of resources on which the development of agriculture and the prosperity of local communities depend [23,24]. Hence, organic farming (OF), which is most often supported with public funds, can be an alternative to conventional agriculture in N2K areas. OF is an on-farm production management system that is closely linked to the quality of the natural environment, as in addition to food production, it is a protective factor for all components of this environment [25].

The issue of environmental protection is progressively becoming a concern for increasingly wider social and professional groups, mainly including farmers. When designating N2K areas, only environmental benefits are taken into account [26]. For this reason, this process has often met with public disapproval, giving rise to a number of conflicts [27]. This may result in declining public support for the establishment and operation of subsequent PAs despite having made global commitments to increase the scale and effectiveness of natural environment protection [2]. However, the establishment of PAs is often in conflict with the needs of local communities, and the introduction of amendments into the legislation not only results in the lack of commitment to enforcing legal rules but also in the downgrading, reducing and loss of legal protection for an entire protected area, i.e., degazettement [28]. This is due to the fact that the inhabitants are rarely allowed to lead non-modified versions of their traditional lifestyles in PAs. The conflicts and problems related to the functioning of local communities in PAs are very common in the modern history of nature conservation but only recently have become an important contribution to public debate. It not only involves local communities living in PAs and in their vicinity but also the naturalists’ circles calling for providing the areas where further endangered plant or animal species or landscape occur with protection.

This also applies to agricultural production. According to legal provisions, there are certain formal restrictions on the development of conventional intensive agriculture, while alternative agriculture is promoted. The latter, in the absence of institutional support, can pose a considerable challenge for farm owners. However, this is the responsibility of farmers, who are financial decision makers, to deal with multi-faceted stressful situations, both in farm management and in family life. Due to the complex dynamics and nature of the agricultural activity and the interdependence with the family, farmers may encounter major challenges associated with financial disruptions that reduce life satisfaction [29]. Satisfaction with one’s life is a cognitive rating of human life quality and is thus used as an indicator of a subjective general feeling or happiness of individuals and their families [29,30,31]. It is therefore important to gain economic satisfaction with the activities pursued, and OF can be an alternative to conventional agriculture in PA.

Poland is among the countries with one of the highest biodiversity levels in the EU. The location of Poland in the transitional area between continental and Atlantic climates, the variety of geographical regions and the lack of large natural boundaries on the West and East of the country are favourable for biodiversity. Moreover, the economic development of Poland and the resulting landscape transformations were hampered for a long time due to the socio-economic conditions [32]. In Poland, there are approximately 70,000 representatives of living organisms, including approximately 3000 vascular plants species and 33,000–47,000 animal species. It is worth stressing that the high biodiversity in Poland is an effect of the extensive, conventional farming carried out for many years, a high proportion of permanent grassland and the occurrence of semi-natural areas, including marshy ones, a large portion of which is currently located within N2K areas. Almost half of the plant assembly types found in Poland have their habitats in rural areas [33,34].

Over the past two decades, OF has experienced rapid growth on a global scale [35]. The area under organic cultivation and the consumption of food produced under the organic system has been on the increase [36]. This particularly applies to Europe, as the top ten countries with the highest consumption of organic products per capita, at the global level, including as many as eight European countries, of which six are EU Member States [37]. In recent years, OF in the EU has been developing quite rapidly. In 2000, the number of organic farms amounted to more than 135,000 and increased to over 344,000 in 2019. The area under cultivation in the EU has been on a rapid increase. The 2019 data for the EU show that the area of agricultural land under the organic system has reached a value of approximately 14,600,000 hectares (16,500,000 hectares throughout Europe). The proportion of organic agricultural land in the EU is considerably greater than in most countries and regions of the world and amounts to 8.1% (Europe: 3.3%; approximately 1.5% of agricultural land worldwide, i.e., 72,300,000 ha in 2019, are under organic cultivation). It is worth mentioning that the European Commission has set a target of achieving the 25% proportion of the area under organic cultivation by the year 2030, which can be linked to the environmental policy decisions related to the functioning of OF in PAs. Moreover, the retail sales values of organic food products in the EU have increased to EUR 41.4 billion (Europe: EUR 45 billion) and the consumption per capita to EUR 84 (Europe: EUR 56). It should be noted that the year 2020 and the COVID 19-related crisis were remarkable to the organic food sector. While the consolidated data for 2019 show a steady upward trend for both organic production and the market, in 2020, the market grew much faster than in the previous years because consumers turned to wholesome and wellness products and paid more attention to preventive health care [38,39].

In Poland, the main factor of the development of organic farms was, for a long time, the high level of subsidisation of this production system [40]. Unfortunately, this translates into an increase in the domestic supply of organic food only to a limited extent [41]. The area of agricultural land under the organic production system in the years 2000–2013 increased over 30-fold to almost 670,000 ha. In 2014, the trend began to reverse, and in 2019, this area was more than 507,000 ha (the proportion of organic agricultural land in total land decreased from 4.7% in 2012 to 3.5% in 2019). The long-term trend in the number of organic farms is similar. In 2000, over 1400 organic farms operated, and, with rapidly growing dynamics, the number amounted to almost 26,000 in 2012, after which the trend of changes reversed. In 2019, over 18,000 certified organic farms were recorded. The proportion of organic farms in Poland is approximately 1.8% of the total number of agricultural farms. The major cause of the regression was the changes in OF support system under the CAP (a reduction in support rates and an increase in requirements conditioning the receipt of subsidies). This is paradoxical in the situation where the demand for organic food is growing, consumers’ spending for this purpose increases, and there are more and more business entities operating in distribution and processing [42,43].

The long-term prosperity of the Earth is primarily determined by the condition of ecosystems and the conservation of their goods and services. However, biodiversity is under severe threat in agricultural landscapes. The intensification and specialisation of agriculture have resulted in a decline in biodiversity and other environmental problems in agri-ecosystems. In view of the concerns about the adverse impact of agriculture on the environment, various governments have been considering the conservation of biodiversity at the farm level [44]. Therefore, environmental and nature protection programmes were developed to counteract these adverse phenomena [45]. Currently, PAs cover almost 13% of the Earth’s surface, yet this form of protection often implies the exclusion of farmers [46]. This is why the agricultural production methods need to be sustainable in economic, environmental and social terms [45]. This is due to the fact that farmers interact directly with the natural ecosystem at the farm level [47] and are therefore able to help in the protection of genetic resources on their agricultural land and the surrounding areas [48]. In order to encourage farmers to protect the environment and take measures aimed at environmental conservation, the EU applies the system of subsidisation, including financial support for farmers’ voluntary participation in agri-environmental schemes (AES). The AES (currently, agri-environmental and climate measures) were introduced into the EU’s Common Agricultural Policy (CAP) in the mid-1980s as an option for the Member States and were a compulsory component of the Member States’ agricultural policy. The AES, in addition to the cross-compliance requirements, are the main instrument used by the EU to strive to satisfy public demand for the environmental services provided by agriculture [46]. However, farmers’ involvement in measures aimed at preserving biodiversity on the farm is determined by many factors [45]. In order to plan an effective intervention that protects biodiversity on the farm, it is necessary to identify the factors determining these measures [44].

The need to protect nature limits human activities, including those related to agriculture. N2K sites, however, are not closed areas in which only bans and restrictions are in place. Regular, rational farming, tourism, or activities in other sectors can be carried out as part of HNV farming, but the fundamental condition is to limit the negative impact on the natural values of the area. If the need arises to restrict a certain type of economic activity in order to adjust to the requirements for an N2K area protection, entrepreneurs or farmers may receive compensation for the lost income arising from the restrictions introduced. This is why, among the incentives arising from farming in N2K areas, financial benefits received under the AES can primarily be distinguished. These are the main instruments of national Rural Development Plans, necessary for the preparation and implementation of biodiversity conservation in PAs, where, e.g., agricultural activities are carried out by the European Union Member States.

This article is an empirical study of the differences in perceiving the possibilities of farm functioning in a PA by organic and conventional farm owners. Therefore, the basis for the considerations and analyses in the publication is the thesis, according to which OF may be an alternative to conventional farming, sustain the development of rural areas and mitigate the effects of restrictions. It was examined whether this could be the actual path to improving farmers’ living conditions in the context of the legal protection of naturally valuable areas. To this end, a construct that is perceived as a subjective rating of the farm’s financial situation was introduced. It is defined as the “actual opportunities to earn an income from agricultural activities by placing emphasis on a farm as an economic unit which serves to ensure an income for a farmer’s family”. The challenges facing the N2K network management were described widely in the literature, with a particular focus on all kinds of socio-economic conflicts between different stakeholder groups that accompany this form of nature conservation [27,49]. This study supplements the gap in the literature through an analysis of the opinions on farmers’ satisfaction with economic outcomes of the farms they operate in PAs, including in N2K areas, under different production systems, i.e., OF and conventional farming. The opinions were gathered by means of a survey based on selected indicators closely related to farmers’ responses. For the analysis of statistical significance, the methodology of cross-tabulation examination based on log-linear analysis was applied. In this context, the opinions of organic and conventional farm owners on the satisfaction of running a farm operating in N2k areas were analysed. In response, we received opinions from farmers in the N2k area “Biebrza Valley” located in Poland. The aim of the study was to analyse the differences in the opinions expressed by organic and conventional farm owners (1) on the level of their satisfaction with their own farm, (2) on whether the farming system (organic or conventional) has an effect on the level of connection of the farm with the market under the conditions of operating in a PA and on whether organic farm owners (3) perform better on the market than conventional farm owners, in a situation where PAs can be a barrier to functioning for the latter.

## 2. Materials and Methods

### 2.1. Area Description—A Case Study

The N2K “Dolina Biebrzy” (“Biebrza Valley”) PLH2000008 area is located in Podlaskie Voivodeship in Augustów, Białystok, Grajewo, Łomża, Mońki, Sokółka and Zambrów counties. It was established in 2008 on an area of 121,206.2 ha. The area was designated based on the occurrence of 16 habitats listed in Annex I to Council Directive 92/43/EEC and 38 bird species listed in Annex I to Council Directive 79/409/EEC. Moreover, in the area concerned, the following organisms listed in Annex II to Council Directive 92/43/EEC are found: 5 mammal species, 2 amphibian and reptile species, 5 fish species, 3 invertebrate species and 6 plant species (Natura 2000—A standard data form for special protection areas (SPA), areas meeting the criteria for sites of Community importance (SCI) and for special areas of conservation (SAC); https://www.biebrza.org.pl/plik,378,soo-dolina-biebrzy.pdf (accessed on 27 July 2021)). In addition, in the “Biebrza Valley” area, a number of other valuable species not mentioned in the above Annexes live, of which the most valuable one is the elk [50,51].

Agricultural land resources in the Biebrza River Valley are characterised by a low Agricultural Production Space Valuation Ratio and a relatively large proportion of poor soils (except peat soils under permanent grasslands) [52,53]. Farming in this location is hampered by the low availability of arable land situated in marshy areas and their generally long distance from the farm, low quality of feed, the fragmentation of land and the rather long distance of farms from service outlets and facilities supplying means of production. Another barrier to the agricultural function implementation is the poorly developed technical infrastructure [54]. In the context of environmental protection in the “Biebrza Valley” area, it is extremely important that the protection measures imitate the treatments applied in an extensive agricultural economy, as they supported the formation and maintenance of non-forest ecosystems of the Biebrza River Valley over several hundred years [55].

Agricultural farms specialise primarily in milk production and fattening cattle breeding as well as the cultivation of maize, potatoes and cereals [56].

### 2.2. Data Gathering

**Figure 1:**
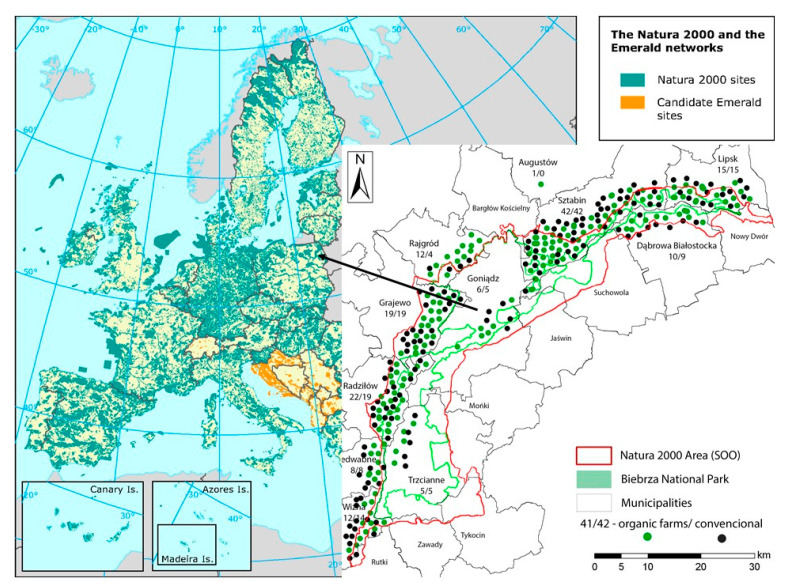
The area of N2K “Biebrza Valley” PLH200008 and the location of farms addressed by the study. Source: background map https://www.eea.europa.eu/data-and-maps/figures/the-natura-2000 accessed on 13 March 2021 and own research.

The study material comprises the results of a survey conducted at the turn of 2016 and 2017, which addressed a total of 292 farmers, including 152 organic farm operators and 140 conventional farm operators, whose areas under cultivation were located within the N2K “Biebrza Valley” PLH200008 area. The study involved all owners of organic farms registered in the organic farm registry system [57] and a homogeneous purposive sample of conventional farm owners who agreed to participate in the study and managed farms in the area under study (Figure 1 shows the locations of farms). In order to minimise the selection bias and maximise the similarities, the study focused on conventional farms located in the vicinity of organic farms, i.e., operating under similar conditions of agroecology, land use, household structure, infrastructure and the distance from markets. The list of conventional farms was established with the help of Biebrza National Park staff, who identified potential respondents. A total of 227 farms were selected, of which 140 owners (61.7% of the defined population) agreed to participate in the study.

Prior to conducting the proper field research, a preliminary study was conducted using pilot questionnaires, which helped prepare the final research tools. Ultimately, two structured questionnaires were developed (one addressed to organic farm owners and another to conventional farm owners). The questionnaire included questions concerning the characteristics of farm owners and their families, the characteristics of farms, information on the functioning in the food raw material market and the perception of the effects of PAs on farms’ activities.

### 2.3. Data Analysis

For the analysis of the results obtained, indicators closely related to the farmers’ opinions were selected [58,59]. The responses were thoroughly checked and coded for the purposes of statistical analysis. The data were entered into the statistical package Statistica v. 13. In order to examine the empirical data reliability, a consistency analysis was conducted using Cronbach’s alpha [60,61], which enabled the assessment of the internal consistency, i.e., how closely the components are linked within a structure, taking into account all limitations of this indicator [62]. The Cronbach’s alpha values for all variables are higher than the recommended level of 0.6, which confirms the reliability of the scales used [63]. A χ^2^ distribution consistency test, which checks whether the distribution of responses to a particular question differs from the random distribution, was also conducted. This test allows the statistical significance to be checked. Table A5 provided in Appendix A contains the definitions of variables, descriptive statistics, Cronbach’s alpha values and the test of compatibility of the variables used in the analysis.

For the analysis of the data collected using structured questionnaires, a variety of statistical methods and techniques were applied. One of the basic data analysis methods is to examine cross tabulations or contingency tables, and a more sophisticated way of perceiving the cross tabulations is the log-linear analysis. This enabled the testing of the statistical significance of the impact of different factors that were entered into a cross tabulation (e.g., production system, gender, opinion, etc.) and their interactions. Correspondence analysis is a descriptive and exploratory technique designed for fourfold and contingency tables containing certain measures of correspondence between rows and columns. This type of analysis is gaining a lot of popularity, particularly in research into social science issues, where variables often cannot be represented differently than on a nominal scale. The results provide information similar to that offered by factor analysis techniques and enable the examination of the structure of the qualitative variables included in Table A5 (Appendix A). By using the χ^2^ test of independence, it was checked as to whether the agricultural production system on a farm had an effect on the opinions expressed by farm owners under the conditions of farm functioning in a PA.

## 3. Results

### 3.1. The Respondents’ and Variables’ Profile

A very important component of making the decision to run an agricultural farm under an organic production system is the farmers’ characteristics, i.e., gender, age and educational background (Table A1). In the study population, males were dominant, both among organic and conventional farm owners. However, it is worth noting the poor correlation between the agricultural production system and gender, although research has demonstrated that two times more women were organic farm owners, as compared to conventional farm owners, which is confirmed by the test probability value *p* being lower than 0.05.

The respondents’ mean age ranged from 35 to 54 years and was, on average, almost 47 years for organic farmers. Conventional farm owners were, on average, one year younger. The analysis demonstrated a significant result of the χ² test, which means that the observed quantities differ significantly from the expected quantities. However, no correlation was observed between the production system applied on farms and their owners’ age.

The educational level was measured on a four-point scale ranging from primary education to tertiary education. Among the respondents, most people had a secondary education, with more organic farmers with such educational background (over 55%) than conventional farmers (over 47%). As regards tertiary education, conventional farmers were slightly dominant (10.22% vs. 8.61% for organic farmers). However, no statistically significant difference was found in the education level (*p* > 0.05). Therefore, no correlation between the educational background level and the agricultural production system was observed either.

### 3.2. Economic Situation of the Farm

It follows from the information provided in Table A2 that there is a statistically significant correlation between the respondents’ satisfaction with operating a farm and the production system, which is indicated by the test probability value *p* being lower than 0.05. The analysis demonstrated that this correlation was relatively poor (φ-0.145) and implied that conventional farmers were slightly more satisfied with the operation of their farms. There were over 86% of satisfied conventional farm owners and over 75% organic farm owners. As for the latter, their opinions primarily arose from the too slow development of the market for organic food raw materials. At the same time, the level of subsidies in Poland under the organic system does not compensate for both the difficulties and higher cultivation costs under this system and the relatively low selling prices and problems with the sales market.

Satisfaction with business activities pursued has an effect on decisions concerning the future of the farm. Analysis of the opinions on the planned changes showed no statistically significant difference between the position of organic farm owners and that of conventional farm owners (*p* > 0.05). It is worth emphasising, however, that among the respondents, almost half of organic farm owners (and slightly fewer conventional farmers) expressed willingness to introduce changes on their farms. The changes indicated by organic farm owners included the purchase of land (34.21%), livestock (13%) and agricultural machinery (7%); the upgrading and construction of farm buildings (3%); and taking-up non-agricultural activities on the farm (e.g., agritourism). Four farmers declared they were going to change the production system because, in their opinion, OF failed to bring the expected financial benefits. Conventional farmers indicated the purchase of land (23.57%), livestock (17.14%) and agricultural machinery (5%) and declared their willingness to upgrade or construct farm buildings (4.29%) and to commence processing of food raw materials on the farm (two respondents).

The level of satisfaction with agricultural activities can be determined by the production resources owned. For agricultural farms, it is primarily the resources of land necessary for production purposes. Good production results include financial benefits and, at the same time, satisfaction with the activities pursued, which has an effect on the future of the farm and the decisions made by the owner in this respect. In the area under study, the average area of an organic farm was 22.41 ha (median of 18.56 ha; a minimum of 3.81 ha; a maximum of 193 ha; SD of 21.02), while the area of a conventional farm reached a value of 25.03 ha (median of 21.64 ha; a minimum of 5 ha; a maximum of 115 ha; SD of 14.28). Statistical analysis shows the occurrence of a relatively weak relationship between the production system and the farm’s area (φ = 0.201), which is indicated by the test probability value *p* being lower than 0.05. It is worth noting that among organic farms, there were as many as four times more farms with an area of more than 100 ha. Nevertheless, conventional farms were dominant within the 30–50 ha range, which strongly affected the average results.

The financial situation considered in terms of effects should be defined as the degree to which common, socially acceptable consumption needs are satisfied with goods and services that are paid or have a monetary market value. This is why the respondents were asked to compare their financial situation to the average earnings in Poland, which in the years 2016/2017 amounted to USD 968.39 (PLN 4047.21) [64]. The level of average monthly disposable income per capita was USD 352.93, which, rounded to the nearest Polish zloty (PLN), amounted to PLN 1475 [65] at the USD exchange rate of PLN 4.1793 as of the last day of 2016 [66]. Analysis indicated that the subjective assessment of the financial situation was not determined by the production system on the farm. The value of Pearson’s χ^2^ statistic was 4.7 and *p* = 0.456. This indicates that there is no statistically significant difference in responses provided to this question among the owners of both organic and conventional farms. It should be noted, however, that the majority of respondents indicated that the financial situation of the farm was good or very good. Only a small percentage of respondents from both groups considered it to be poor or very poor.

Analysis of farm income sources demonstrated a statistically significant difference between the opinions of organic and conventional farm owners. The test probability for the χ^2^ test was lower than the assumed significance level of 0.05 and amounted to 0.001. This shows that the production system on a farm differentiates the origin of income. Admittedly, the strength of association is relatively small (φ 0.236), yet it can be observed that in more than 53% of conventional farms, income is only earned from agricultural production, while for organic farms, it was true for only a third of farms (Table A3). On organic farms, almost half of the income was generated partly by agricultural production and partly by work outside the farm, while on conventional ones, work outside the farm accounted for less than a third of the declared income sources.

Data analysis showed no statistically significant correlation between the production system and the operation in the “Biebrza Valley” N2K site. According to the majority of respondents’ opinions (over 67% among organic farmers and 62% among conventional ones), they felt no impact of a legally protected area on the business activities they pursued. Only a third of farmers (from both groups) noted the positive effect, while only five conventional farm owners (3.57%) indicated adverse effects of farm location within an N2K area. According to the farmers, the positive effect is, primarily, the payments, while the negative one is the barriers to the development of intensive production.

The main purpose of a business entity is to persist and develop, while that of its owner is to earn profit. Both purposes are interrelated, as a positive financial result enables investments that guarantee the persistence and development of the enterprise. Therefore, the subjective satisfaction with the business activity pursued is related to the financial benefits that the farm owner can derive, which in turn are related to the conditions determined by both the market and regulations.

Data analysis (Table A4) demonstrated that there was a statistically significant dependency between the production system and the sales of agricultural raw materials produced on the farm (φ 0.204), which is indicated by the probability level *p* being lower than 0.05. Of all the respondents, it is organic farm owners that declared greater problems with sales. More than 43% sold only a small portion of their products, while as many as 81.3% indicated constant difficulties in finding a sales market for organic raw materials. Importantly, organic farm owners claimed that they had to sell a portion of organic raw materials as conventional ones (for lower prices) due to the lack of sales market.

Regarding the farmers operating conventional farms, most of them (64%) sold more than half, or almost all, with no problems whatsoever. Less than a third of them declared that they sold a small portion of their produce, while approximately a fourth of conventional farm owners declared having constant difficulties in finding a sales market.

In addition, the profitability of the pursued business activities showed a statistically significant correlation (φ 0.274; *p* < 0.05). Among the respondents, significantly more conventional farmers claimed that the type of production on the farm and the sales of food raw materials brought them financial benefits. Regarding organic farmers, there were more respondents who believed that their activities were unprofitable or that the return-on-investment rate was low.

A very important aspect of functioning on the market is the distribution channels. Unfortunately, most respondents, i.e., more than 82% of organic farmers and more than 60% of conventional farmers, indicated incidental sales. Nevertheless, the statistically significant relationship (φ 0.323; *p* < 0.05) shows that more conventional farm owners have concluded permanent sales contracts than organic farm owners. This may be due to the fact that almost five times more conventional farmers made use of advisory institutions’ services and attempted to introduce innovations. Regarding the farmers participating in the study, the vast majority were not engaged in processing activities.

## 4. Discussion

Until the middle of the 20th century, the natural values of agricultural land were not appreciated. Nature protection was associated primarily with species protection and spatial forms of protection. On the other hand, the enormous importance of protecting biodiversity in agriculturally used areas for our cultural heritage was not noticed [67,68,69]. Consequently, the intensive development of agriculture worldwide resulted in the loss of numerous valuable ecosystems and thus in the considerable decline in the biodiversity level in rural areas [70,71,72,73,74]. In order to protect biodiversity, the N2K ecological network was established in the EU. The N2K site network programme in Poland has been implemented since 2004, i.e., since the accession to the European Union. According to Habuda [75], as compared to other nature protection forms, an N2K site appears to be a very flexible form of protection. However, farmers under such conditions have limited capacity to make decisions that enable profit maximisation and achieve policy objectives of protecting valuable areas on agricultural land involves additional costs. High financial expenditure may result in farmers ceasing their production. A specific example is naturally valuable permanent grassland that is threatened by a land-use change, abandonment and afforestation [76,77,78,79]. It is therefore essential to implement programmes that support farmers financially, at least for a certain period, which will compensate for keeping the area under extensive agricultural use, including under the organic system [80]. According to Hochkirch et al. [81], it is also necessary to allocate more funds on education that will raise public awareness (especially among rural area users) about the need for biodiversity conservation.

The basic objectives of OF are very simple. Firstly, it is to produce agro-food raw materials of high nutritional and agricultural value in sufficient quantities. Secondly, it is to participate in environmental policy, i.e., to take measures to support all life processes occurring in natural systems, instead of attempting to dominate nature [82]. However, for many years there has been a debate as to whether OF reduces the adverse impact on the environment. In general, it is believed that OF promises to solve the problems of growing social costs associated with the negative effect of agriculture on the natural environment and the improvement in animal welfare and food quality, as well as safety. This is mainly due to the fact that the wide range of policy instruments implemented at the European and national levels support the development of OF [83]. It should be noted that there is a growing consensus that OF ensures certain environmental benefits, as compared to conventional farming [84]. In general, OF, in terms of the area, has a lower adverse impact on the environment than conventional farming. However, when comparing product units, the benefits of organic farming are lower than they appear to be [85]. Attention is also increasingly drawn to the role of OF in the rural economy and, in particular, to the potential of OF in rural development [84].

A subjective assessment of the farm’s financial situation is a relevant research issue, as irrespective of changing economic conditions of farming, the determinants associated with the sphere of public awareness cannot be ignored. This is because farmers’ opinions and attitudes have a decisive impact on agricultural reality, and farmers’ satisfaction with farm work can have far-reaching consequences [86]. The production decisions they take are often the outcome of many factors that go beyond the objective economic calculation [87]. However, the subjective significance attributed to work is individual, multi-dimensional and complex [88]. The assessment of the farm’s financial situation is primarily differentiated by the production methods applied: the most modern they are, the higher the rating of the farm’s economic position. The farmers who do not work outside the farm as well as those who specialise in livestock production, also rate their situation much higher [89]. Therefore, satisfaction with financial outcomes achieved on the farm is an important aspect. As for the agricultural sector, the alternative costs are often low, and farmers are forced to accept the level of earnings achieved, which often represent a low income as compared to other sectors [90]. However, in this study, the respondents considered that the financial benefits received from the farm business were good or very good, compared to average earnings in Poland. Such opinions were affected by neither the production system on the farm nor the actual operation in PAs. It should be noted, however, that under the conditions in which the respondents’ farms operated, conventional farm owners were slightly more satisfied, which is primarily due to the access to an extensive and secure sales market. For organic production, it is very difficult to sell raw materials. In the respondents’ opinions, the income is mainly determined by subsidies to agricultural production, including programmes supporting agri-environmental measures, which are available to conventional farm owners as well. This may be due to the relative safety ensured by the system of protective subsidies for agriculture [86], although Besser and Mann [91], who compared the highly competitive, lowly subsidised (conventional) agricultural system with the non-competitive, highly subsidised (organic) system, found that monetary utility did not play a significant role for the satisfaction with work under a subsidised system.

Moreover, in terms of making decisions related to the development on the farm, no difference was observed between the organic and conventional farm owners, as both intend to invest in increasing their production area, the purchase of livestock or equipment and the upgrading of buildings and machinery. Farm owners’ interest in investments in the Biebrza River Valley is also confirmed by a study by Gołębiewska and Stefańczyk [33]. According to these authors, these investments in particular concern farm buildings, livestock and, above all, residential buildings. However, it should be borne in mind that besides the positive environmental effects, the establishment of N2K areas may also significantly change the economic conditions for the users of land located within their boundaries. An example here could be the direct payments under the Common Agricultural Policy, including environmental payments that can, theoretically, significantly increase the price of land [22], which can consequently hinder the expansion of farms. Only a small percentage of organic farm owners did not see a future in continuing production under the organic system, primarily due to the lack of sales market.

Although more than 18,000 registered organic farmers in Poland account for less than 1.8% of the farm population and the area under organic cultivation amounts to half a million hectares, with a total proportion in the agricultural land of only 3.5%, the organic product market increased from EUR 1,500,000 in 2004 to approximately EUR 314,000,000 euro in 2019 [38,42]. Despite dynamic changes in the retail market, the primary market of organic farm owners is characterised by limited sales, incidental sales, the lack of long-term contracts and sales of a portion of organic raw materials on conventional food markets. As a result, the agricultural production system may have an effect on the agricultural farm’s performance. This is why organic farm owners very often have more problems with sales. This primarily results from the fragmentation and poor concentration of supply on organic farms, the small number of processing operators and the poorly developed distribution network based mainly on imported products [92]. Not without significance is the fact that on organic farms, many owners supplement their income outside the farm [93]. On the other hand, income on conventional farms is primarily earned from the sales of agricultural raw materials, and this activity is often profitable. Both the scale of production and the production results achieved are very important. On organic farms, these are lower than for conventional production. With a limited sales market, price premium (where successfully obtained) does not guarantee adequate income. Additionally, with increasing market supply, there is a change in the trend of price premiums for organic food products, which reduces revenue with the sales levels remaining unchanged [41]. However, despite the problems affecting the activities of farms operating within the N2K network, research indicated no differences and the significance of the farming system on the level of farms’ linkage to the market under the conditions of operating in PAs.

The establishment of PAs requires, on the one hand, properly constructed legislation in order to reconcile human activities and environmental protection and, on the other hand, the local community’s awareness of both the disadvantages and benefits of this solution and the consent for it. For example, land protected under the N2K scheme is automatically subject to the non-degradation principle [9] and can therefore reduce farmers’ flexibility in the use of resources [22]. It should be stressed, however, that numerous studies indicate the opposite direction of impact, i.e., no noticeable effect on economic activity [7,94,95,96,97,98]. The results of our study show that most respondents declared that they did not notice the impact of PAs on their business. Those respondents that did notice this impact indicated its both positive (e.g., financial support) and negative aspects (e.g., restrictions on investments, including in the intensification of production). On the other hand, the high level of satisfaction (similar among organic and conventional farmers) with the effects of farm activities shows that organic farm owners, despite greater “facilitations” in the form of higher subsidies, do not cope better than conventional farm owners, even though PAs can, in theory, form a significantly greater barrier to the functioning and development of conventional farms.

## 5. Conclusions

However, the policy to protect naturally valuable agricultural land involve costs and the owners of farms located in PAs have limited ability to make decisions enabling profit maximisation. The literature provides theoretical and empirical explanations about the effects of agriculture on the natural environment while relatively rarely discussing the issue of the effects of PAs on economic activity, including agriculture. This study supplements this knowledge based on the opinions of owners of conventional and organic farms located within PAs. This study fills a gap in the literature on the subject by answering the question as to what production system on a farm operating in a PA is, in their owners’ opinions, better in terms of economic performance results. The study results indicated that in terms of satisfaction with the economic performance of their farms, there is no major difference between the opinions expressed by organic and conventional farm owners. Equally important is that in research, quantitative analyses are not necessarily as good as the particular persons’ views and satisfaction level. This is important because satisfaction with economic outcomes and the farm owners’ acceptance are the crucial aspect of further agricultural use of land and the effective protection of natural resources.

It should also be stressed that in order to generalise our findings, a much larger sample of agricultural farms from different regions would be needed. An important aspect of the assessment of farms’ operation in PAs is the multi-criteria analysis prepared by economists, naturalists, farmers, agricultural production technologists, etc. This is due to the fact that publicly supported organic farming can be less of a burden to ecosystems than extensive, conventional farming. Proper understanding of the problem can contribute to both an improvement in the effectiveness of conservation measures and a reduction in (financial and alternative) economic costs. It is important to create an integrated support package for farmers operating in N2K areas, which will ensure the profitability of the organic agricultural model (a compensation scheme). That is crucial for the protection of PAs and, secondly, refers to the specific measures necessary for the protection of key habitats and species. The primary aim in this respect is to ensure the cost-effectiveness of organic farms. Building the capacity of the agricultural infrastructure (and the farmer) in this regard and increasing the market income of organic farms may help in achieving this objective.

Despite the important findings of this study, there are certain restrictions that must be taken into account when interpreting the results and drawing the main conclusions. Firstly, an assessment of the subjective perception of the quality of life actually includes only one aspect, namely the financial situation arising from the fact of owning a farm. It is important to include objective indicators assessing the level of economic, social and environmental efficiency in a future piece of research. Secondly, even though the opinions explaining the perception of possibilities of farm functioning in N2K areas were examined, the results are not exhaustive. In the increasingly intense debate on the functioning of economic activities, additional factors should be indicated, including primarily the effect of agriculture on PAs, in particular concerning sustainable development. It is also important that future research focuses on gathering quantitative and qualitative data through interviews and focus groups, including key stakeholders. Studies based on mixed methods provide an in-depth understanding of the links between various socio-economic and environmental components. In addition, the “participatory” nature of these qualitative techniques enables more effective communication and the exchange of knowledge on such research findings.

We hope that the results of our empirical study will provide scientists, practitioners and decision makers with important observations and influence the shaping of agricultural policy in this area, not only in Poland. In the PA, including N2K, it is particularly important to maintain agriculture of high natural quality and certified organic farming falls within this category. In this context, farmers’ subjective feelings are of importance. This is because there is a real danger that farmers’ dissatisfaction with carrying out agricultural production under this system will result in it being either abandoned or intensified.

## Data Availability

Not applicable.

## Appendix A

**Table A1:** Individual characteristics of respondents.

	Organic		Conventional		Total
	*n*	%	*n*	%	*n*
Gender					
Female	23	15.54%	10	7.25%	33
Male	125	84.46%	128	92.75%	253
Total	148	100.00%	138	100.00%	286
Pearson’s Chi^2	4.813	df = 1	*p* = 0.028 *		
Phi coefficient	0.130				
Age					
25–34	18	12.00%	20	14.39%	38
35–44	53	35.33%	56	40.29%	109
45–54	54	36.00%	39	28.06%	93
More than 55	25	16.67%	24	17.27%	49
Total	150	100.00%	139	100.01%	289
Pearson’s Chi^2	2.212	df = 3	*p* = 0.530		
Education					
Elementary school	16	10.60%	10	7.30%	26
Vocational school	41	27.15%	48	35.04%	89
High school	81	53.64%	65	47.45%	146
College	13	8.61%	14	10.22%	27
Total	151	100.00%	137	100.00%	288
Pearson’s Chi^2	3.052	df = 3	*p* = 0.384		

* statistically significant (*p* < 0.05).

**Table A2:** Characteristics of farms’ economic situation.

	Organic		Conventional		Total
	*n*	%	*n*	%	*n*
Satisfaction with managing a farm					
Yes	113	75.33%	78	57.35%	140
No	37	24.67%	18	13.24%	55
Total	150	100.00%	136	100.00%	286
Pearson’s Chi^2	6.000	df = 1	*p* = 0.014 *		
Phi coefficient	−0.145				
Planned changes on the farm					
Yes	69	48.94%	58	41.43%	127
No	72	51.06%	82	58.57%	154
Total	141	100.00%	140	100.00%	281
Pearson’s Chi^2	1.599	df = 1	*p* = 0.206		
Farm area					
Up to 5 ha	2	1.32%	1	0.72%	3
5–15 ha	47	31.13%	31	22.14%	78
15–30 ha	83	54.97%	76	54.29%	159
30–50 ha	12	7.95%	28	20.00%	40
50–100 ha	3	1.99%	3	2.14%	6
More than 100 ha	4	2.65%	1	0.71%	5
Total	151	100.01%	140	100.00%	291
Pearson’s Chi^2	11.725	df = 5	*p* = 0.039 *		
Phi coefficient	0.201		V Craméra	0.201	

* statistically significant (*p* < 0.05).

**Table A3:** Respondents’ opinions on their farm’s financial situation.

	Organic		Conventional		Total
	*n*	%	*n*	%	*n*
Subjective financial situation assessment					
Very good	20	13.42%	22	15.71%	42
Good	84	56.38%	72	51.43%	156
Satisfactory	7	4.70%	12	8.57%	19
Sufficient	26	17.45%	18	12.86%	44
Poor	11	7.38%	13	9.29%	24
Very poor	1	0.67%	3	2.14%	4
Total	149	100.00%	140	100.00%	289
Pearson’s Chi^2	4.680	df = 5	*p* = 0.456		
Sources of farm income					
Exclusively from agricultural production (more than 90% of income)	51	34.00%	75	53.57%	126
Mainly from agricultural production (more than 50% of income)	24	16.00%	26	18.57%	50
Partially from agricultural production (20–50% of income)	73	48.67%	37	26.43%	110
Income from the farm accounts for a small percentage (up to 20% of income)	2	1.33%	2	1.43%	4
Total	150	100.00%	140	100.00%	290
Pearson’s Chi^2	16.108	df = 3	*p* = 0.001 *		
Phi coefficient	0.236		V Craméra	0.236	
Opinion on the effect of N2K site on farm functioning					
Positive	49	32.89%	47	33.57%	96
Negative	0	0.00%	5	3.57%	5
No impact	100	67.11%	88	62.86%	188
Total	149	100.00%	140	100.00%	289
Pearson’s Chi^2	5.533	df = 2	*p* = 0.063		

* statistically significant (*p* < 0.05).

**Table A4:** Respondents’ opinions on farm functioning.

	Organic		Conventional		Total
	*n*	%	*n*	%	*n*
The sales of produced agricultural raw materials on the market					
I sell nothing	8	5.41%	4	2.86%	12
It is difficult to say, but a small portion	64	43.23%	46	32.86%	110
Almost half of what I produce	17	11.49%	11	7.86%	28
More than half of what I produce	26	17.57%	48	34.28%	74
Almost everything I produce	33	22.30%	31	22.14%	64
Total	148	100.00%	140	100.00%	288
Pearson’s Chi^2	11.955	df = 4	*p* = 0.018 *		
Phi coefficient	0.204		Cramér’s V	0.204	
Profitability of the production carried out					
Unprofitable	27	18.00%	18	12.86%	45
Hardly profitable	82	54.67%	54	38.57%	136
Break-even	11	7.33%	22	15.71%	33
Profitable	22	14.67%	44	31.43%	66
Very profitable	8	5.33%	2	1.43%	10
Total	150	100.00%	140	100.00%	290
Pearson’s Chi^2	21.846	df = 4	*p* = 0.000 *		
Phi coefficient	0.274464		Cramér’s V	0.274464	
Difficulties in selling organic agricultural raw materials					
This is almost always the case for me	113	81.30%			
It depends on what products are concerned, but I often have difficulties	13	9.35%			
Generally, I have no difficulties	13	9.35%			
Total	139	100.00%			
Difficulties in selling conventional agricultural raw materials					
This is almost always the case for me	5	3.55%	34	24.28%	39
It depends on what products are concerned, but I often have difficulties	10	7.09%	16	11.43%	26
Generally, I have no difficulties	126	89.36%	90	64.29%	216
Total	141	100.00%	140	100.00%	281
Pearson’s Chi^2	28.946	df = 2	*p* = 0.000 *		
Phi coefficient	0.321		Cramér’s V	0.321	
Agricultural raw material distribution channels					
I have concluded long-term delivery contracts for everything I can sell	2	1.34%	22	15.71%	24
It depends on the product, but I have concluded an agreement for most of them	7	4.70%	19	13.58%	26
It depends on the product, but I sell only certain products under the long-term contract	9	6.04%	7	5.00%	16
I sell incidentally	123	82.55%	85	60.71%	208
I sell together with other farmers through a producer group	1	0.67%	0	0.00%	1
I sell nothing	7	4.70%	7	5.00%	14
Total	149	100.00%	140	100.00%	289
Pearson’s Chi^2	30.146	df = 5	*p* = 0.000 *		
Phi coefficient	0.323		Cramér’s V	0.323	
Measures to improve product quality					
I believe that I produce raw materials of good quality	94	63.95%	67	47.86%	161
I make use of advisory services, read, watch TV and try to introduce innovations on the farm	14	9.52%	65	46.43%	79
I work under specialists’ guidance, and follow their recommendations	1	0.68%	5	3.57%	6
1 + 2	24	16.33%	3	2.14%	27
1 + 2+3	14	9.52%	0	0.00%	14
Total	147		140		287
Pearson’s Chi^2	70.323	df = 4	*p* = 0.000 *		
Phi coefficient	0.495		Cramér’s V	0.495	
Processing activities					
Yes	4	2.67%	10	7.25%	14
No	146	97.33%	128	92.75%	274
Total	150	100.00%	138	100.00%	288
Pearson’s Chi^2	3.260	df = 1	*p* = 0.071		

* statistically significant (*p* < 0.05).

**Table A5:** Definition, descriptive statistics and Cronbach’s Alpha of the variables.

Variable	Definition	Mean		S.D.		Coefficient of Variation	
		Org	Con	Org	Con	Org	Con
Individual characteristics							
Gender	Female = 1, Male = 2 (χ² = 169.231; df = 1; *p* < 0.001)	1.845	1.928	0.364	0.260	19.707	13.499
Age (years)	Lower than 24 = 1, 25–34 = 2, 35–44 = 3, 45–54 = 4, More than 55 = 5 (χ² = 48.37; df = 3; *p* < 0.001)	3.573	3.482	0.907	0.943	25.395	27.081
Education	Elementary school = 1, Vocational school = 2, High school = 3, College = 4 (χ² = 137.583; df = 3; *p* < 0.001)	2.603	3.550	0.792	0.851	30.442	23.970
α		0.861	0.794				
Family characteristics							
α		0.711	0.751				
Characteristics of farms’ economic situation							
Satisfaction with managing a farm	Yes = 1; N0 = 2; (χ² = 38.189; df = 2; *p* < 0.001)	1.927	1.721	0.868	0.892	45.038	51.835
Planned changes on the farm	Yes = 1; No = 2 (χ² = 108.308; df = 1; *p* < 0.001)	1.511	1.586	0.502	0.494	33.209	31.176
Farm area	up to 5 ha = 1; 5–15 ha = 2; 15–30 ha = 3; 30–50 ha = 4; 50–100 ha = 5; more than 100 ha = 6 (χ² = 390.134; df = 5; *p* < 0.001)	2.861	3.029	0.864	0.777	30.214	25.651
α		0.784	0.715				
Respondents’ opinions on their farm’s financial situation							
Subjective financial situation assessment	Very good = 1; Good = 2; Satisfactory = 3; Sufficient = 4; Poor = 5; Very poor = 6 (χ² = 312.848; df = 5; *p* < 0.001)	2.510	2.550	1.183	1.283	47.143	50.297
Sources of farm income	Exclusively from agricultural production (more than 90% of income) = 1; Mainly from agricultural production (more than 50% of income) = 2; Partially from agricultural production (20–50% of income) (20–50%) = 3; Income from the farm accounts for a small percentage (up to 20% of income) (do 20%) = 4 (χ² = 130.579; df = 3; *p* < 0.001)	2.173	1.757	0.925	0.897	42.562	51.026
Opinion on the effect of N2K site on farm functioning	Positive =1; Negative = 2; No impact = 3 (χ² = 173.820; df = 2; *p* < 0.001)	2.342	2.293	0.943	0.941	40.250	41.026
α		0.698	0.656				
Respondents’ opinions on farm functioning							
The sales of produced agricultural raw materials on the market	I sell nothing = 1; It is difficult to say, but a small portion = 2; Almost half of what I produce = 3; More than half of what I produce = 4; Almost everything I produce = 5 (χ² = 104.361; df = 4; *p* < 0.001)	3.081	3.400	1.312	1.234	42.582	36.289
Profitability of the production carried out	Unprofitable = 1; Hardly profitable = 2; Break-even = 3; Profitable = 4; Very profitable = 5 (χ² = 159.414; df = 4; *p* < 0.001)	2.347	2.700	1.099	1.091	46.834	40.401
Difficulties in selling organic agricultural raw materials	This is almost always the case for me = 1; It depends on what products are concerned, but I often have difficulties = 2; Generally, I have no difficulties = 3 (χ² = 189.918; df = 3; *p* < 0.001)	1.281		0.626		48.875	
Difficulties in selling conventional agricultural raw materials	This is almost always the case for me = 1; It depends on what products are concerned, but I often have difficulties = 2; Generally, I have no difficulties = 3 (χ² = 240.562; df = 2; *p* < 0.001)	2.858	2.400	0.440	0.855	15.411	35.623
Agricultural raw material distribution channels	I have concluded long-term delivery contracts for everything I can sell = 1; It depends on the product, but I have concluded an agreement for most of them = 2; It depends on the product, but I sell only certain products under the long-term contract = 3; I sell incidentally = 4; I sell together with other farmers through a producer group = 5; I sell nothing = 6 (χ² = 644.612; df = 5; *p* < 0.001)	3.906	3.307	0.747	1.319	19.136	39.881
Measures to improve product quality	I believe that I produce raw materials of good quality = 1; I make use of advisory services, read, watch TV and try to introduce innovations on the farm = 2; I work under specialists’ guidance and follow their recommendations = 3; 1 + 2=4; 1 + 2 + 3 = 5 (χ² = 290.056; df = 4; *p* < 0.001)	2.170	1.600	1.917	0.666	88.356	41.607
Processing activities	Yes = 1; No = 2 (χ² = 234.722; df = 1; *p* < 0.001)	1.973	1.928	0.162	0.260	8.192	13.499
α		0.714	0.784				

## References

[1] Juffe-Bignoli D., Burgess N.D., Bingham H., Belle E.M.S., de Lima M.G., Deguignet M., Bertzky B., Milam A.N., Martinez-Lopez J., Lewis E. (2014). Protected Planet Report: Tracking Progress towards Global Targets for Protected Areas.

[2] Watson J.E.M., Dudley N., Segan D.B., Hockings M. (2014). The Performance and Potential of Protected Areas. Nature.

[3] Ma B., Zhang Y., Hou Y., Wen Y. (2020). Do Protected Areas Matter? A Systematic Review of the Social and Ecological Impacts of the Establishment of Protected Areas. Int. J. Environ. Res. Public. Health.

[4] Dudley N., Stolton S., Belokurov A., Krueger L., Lopoukhine N., MacKinnon K., Sandwith T., Sekhran N. (2010). Natural Solutions: Protected Areas Helping People Cope with Climate Change.

[5] IUCN (2012). The Role of Protected Areas in Regard to Climate Change Scoping Study, Georgia.

[6] Jones N., Malesios C., Ioannidou E., Kanakaraki R., Kazoli F., Dimitrakopoulos P.G. (2018). Understanding Perceptions of the Social Impacts of Protected Areas: Evidence from Three NATURA 2000 Sites in Greece. Environ. Impact Assess. Rev..

[7] Schirpke U., Scolozzi R., Da Re R., Masiero M., Pellegrino D., Marino D. (2018). Recreational Ecosystem Services in Protected Areas: A Survey of Visitors to Natura 2000 Sites in Italy. J. Outdoor Recreat. Tour..

[8] Soler Luque Z., Kostecka J. (2018). Biodiversity Loss, the Causes, the State and Basic Form of Nature Protection in Spain and Poland. J. Sustain. Dev..

[9] EC (1992). Council Directive 92/43/EEC of 21 May 1992 on the Conservation of Natural Habitats and of Wild Fauna and Flora.

[10] Evans D. (2012). Building the European Union’s Natura 2000 Network. Nat. Conserv..

[11] EC (2018). Farming for Natura 2000. Guidance on How to Support Natura 2000 Farming Systems to Achieve Conservationobjectives, Based on Member States Good Practice Experiences.

[12] EC (2020). Natura 2000 Barometer. Nat. Biodivers. Newsl. Eur. Comm..

[13] Müller A., Schneider U.A., Jantke K. (2018). Is Large Good Enough? Evaluating and Improving Representation of Ecoregions and Habitat Types in the European Union’s Protected Area Network Natura 2000. Biol. Conserv..

[14] ENRD (2018). EU Rural Review.

[15] EC (2009). Directive 2009/147/EC of the European Parliament and of the Council of 30 November 2009 on the Conservation of Wild Birds.

[16] Cooper T., Hart K., Baldock D. (2009). Provision of Public Goods through Agriculture in the European Union.

[17] de Groot R.S., Alkemade R., Braat L., Hein L., Willemen L. (2010). Challenges in Integrating the Concept of Ecosystem Services and Values in Landscape Planning, Management and Decision Making. Ecol. Complex..

[18] Martino S., Muenzel D. (2018). The Economic Value of High Nature Value Farming and the Importance of the Common Agricultural Policy in Sustaining Income: The Case Study of the Natura 2000 Zarandul de Est (Romania). J. Rural Stud..

[19] Power A.G. (2010). Ecosystem Services and Agriculture: Tradeoffs and Synergies. Philos. Trans. R. Soc. B Biol. Sci..

[20] Swinton S.M., Lupi F., Robertson G.P., Hamilton S.K. (2007). Ecosystem Services and Agriculture: Cultivating Agricultural Ecosystems for Diverse Benefits. Ecol. Econ..

[21] Beaufoy G., Marsden K. (2010). CAP Reform 2013: Last Chance to Stop the Decline of Europe’s High Nature Value Farming?.

[22] Koemle D., Lakner S., Yu X. (2019). The Impact of Natura 2000 Designation on Agricultural Land Rents in Germany. Land Use Policy.

[23] Jackson L.E., Pascual U., Hodgkin T. (2007). Utilizing and Conserving Agrobiodiversity in Agricultural Landscapes. Agric. Ecosyst. Environ..

[24] Hassan R.M., MEA (2005). A Report of the Millennium Ecosystem Assessment. Ecosystems and Human Well-Being.

[25] Markuszewska I., Kubacka M. (2017). Does Organic Farming (OF) Work in Favour of Protecting the Natural Environment? A Case Study from Poland. Land Use Policy.

[26] Halada L., Evans D., Romão C., Petersen J.-E. (2011). Which Habitats of European Importance Depend on Agricultural Practices?. Biodivers. Conserv..

[27] Maczka K., Matczak P., Jeran A., Chmielewski P.J., Baker S. (2021). Conflicts in Ecosystem Services Management: Analysis of Stakeholder Participation in Natura 2000 in Poland. Environ. Sci. Policy.

[28] Mascia M.B., Pailler S. (2011). Protected Area Downgrading, Downsizing, and Degazettement (PADDD) and Its Conservation Implications. Conserv. Lett..

[29] Heo W., Lee J.M., Park N. (2020). Financial-Related Psychological Factors Affect Life Satisfaction of Farmers. J. Rural Stud..

[30] Diener E., Emmons R.A., Larsen R.J., Griffin S. (1985). The Satisfaction With Life Scale. J. Pers. Assess..

[31] Pavot W., Diener E., Diener E. (2009). Review of the Satisfaction With Life Scale. Assessing Well-Being: The Collected Works of Ed Diener.

[32] Świtek S., Jankowiak Ł., Rosin Z.M., Sawinska Z., Steppa R., Takacs V., Zbyryt A., Tryjanowski P. (2017). Jak Zachować Wysoki Poziom Bioróżnorodności Na Obszarach Rolniczych w Polsce? Identyfikacja Najważniejszych Problemów Badawczych. Wieś Rol..

[33] Gołębiewska B., Stefańczyk J. (2018). Investments on Protected Areas on the Example of Biebrza National Park. Rocz. Nauk. Stowarzyszenia Ekon. Rol. Agrobiz..

[34] Gotkiewicz W., Wiśniewska A. (2018). Threats to Biodiversity in Natura 2000 Sites on the Example of the Region of Warmia and Mazury. Environ. Prot. Nat. Resour..

[35] Willer H., Meier C., Schlatter B., Dietemann L., Kemper L., Trávníček J., Willer H., Trávníček J., Meier C., Schlatter B. (2021). The World of Organic Agriculture 2021: Summary. The World of Organic Agriculture. Statistics and Emerging Trends 2021.

[36] Bryła P. (2016). Organic Food Consumption in Poland: Motives and Barriers. Appetite.

[37] Brodzińska K. (2018). The Ecologization of Agriculture in Aspect of FinancialSupport Policy (Ekologizacja Rolnictwa w Aspekcie Polityki Finansowego Wsparcia). Probl. World Agric. Rol. Świat..

[38] Trávníček J., Willer H., Schaack D., Willer H., Trávníček J., Meier C., Schlatter B. (2021). Organic Farming and Market Development in Europe and the European Union. The World of Organic Agriculture. Statistics and Emerging Trends 2021.

[39] Willer H., Moeskops B., Busacca E., Brisset L., Gernert M., Schmidt S., Willer H., Trávníček J., Meier C., Schlatter B. (2021). Europe. Organic in Europe: Recent Developments. The World of Organic Agriculture. Statistics and Emerging Trends 2021.

[40] Brodzińska K. (2015). Problems of Biodiversity Conservation in Polish Agriculture. Agroecol. Sustain. Food Syst..

[41] Pawlewicz A. (2020). Change of Price Premiums Trend for Organic Food Products: The Example of the Polish Egg Market. Agriculture.

[42] Pawlewicz A., Brodzinska K., Zvirbule A., Popluga D. (2020). Trends in the Development of Organic Farming in Poland and Latvia Compared to the EU. Rural Sustain. Res..

[43] Schlatter B., Trávníček J., Meier C., Keller O., Willer H., Willer H., Trávníček J., Meier C., Schlatter B. (2021). Current Statistics on Organic Agriculture Worldwide: Area, Operators and Market. The World of Organic Agriculture. Statistics and Emerging Trends 2021.

[44] Maleksaeidi H., Keshavarz M. (2019). What Influences Farmers’ Intentions to Conserve on-Farm Biodiversity? An Application of the Theory of Planned Behavior in Fars Province, Iran. Glob. Ecol. Conserv..

[45] Ahnström J., Höckert J., Bergeå H.L., Francis C.A., Skelton P., Hallgren L. (2009). Farmers and Nature Conservation: What Is Known about Attitudes, Context Factors and Actions Affecting Conservation?. Renew. Agric. Food Syst..

[46] Cavanagh C.J., Benjaminsen T.A. (2015). Guerrilla Agriculture? A Biopolitical Guide to Illicit Cultivation within an IUCN Category II Protected Area. J. Peasant Stud..

[47] Jackson L., van Noordwijk M., Bengtsson J., Foster W., Lipper L., Pulleman M., Said M., Snaddon J., Vodouhe R. (2010). Biodiversity and Agricultural Sustainagility: From Assessment to Adaptive Management. Curr. Opin. Environ. Sustain..

[48] Guillem E.E., Murray-Rust D., Robinson D.T., Barnes A., Rounsevell M.D.A. (2015). Modelling Farmer Decision-Making to Anticipate Tradeoffs between Provisioning Ecosystem Services and Biodiversity. Agric. Syst..

[49] Iojă I.-C., Hossu C.-A., Niţă M.-R., Onose D.-A., Badiu D.-L., Manolache S. (2016). Indicators for Environmental Conflict Monitoring in Natura 2000 Sites. Procedia Environ. Sci..

[50] Borowik T., Ratkiewicz M., Maślanko W., Duda N., Kowalczyk R. (2020). The Level of Habitat Patchiness Influences Movement Strategy of Moose in Eastern Poland. PLoS ONE.

[51] EC Natura 2000-Standard Data-N2K PLH200008 Dataforms. https://natura2000.eea.europa.eu/Natura2000/SDF.aspx?site=PLH200008.

[52] Banaszuk H., Banausz P., Banaszuk H. (2004). Charakterystyka Gleb Biebrzańskiego Parku Narodowego. Kotlina Biebrzańska i Biebrzański Park Narodowy. Aktualny Stan, Walory, Zagrożenia i Potrzeby Czynnej Ochrony.

[53] Gotkiewicz J. (1972). Wyniki Dwudziestoletnich Doświadczeń Na Torfowiskach Wysokich. Wiad Melior.

[54] Bałtromiuk A. (2011). Gospodarcze i Społeczne Aspekty Funkcjonowania Sieci Natura 2000 w Parkach Narodowych.

[55] Mioduszewski W., Gotkiewicz J., Banaszuk H. (2004). Ochrona Walorów Przyrodniczych Doliny Biebrzy. Kotlina Biebrzańska i Biebrzański Park Narodowy. Aktualny Stan, Walory, Zagrożenia i Potrzeby Czynnej Ochrony.

[56] Markowski A. (2020). Uwarunkowania Rozwoju Rolnictwa w Województwie Podlaskim. Stud. Rap. IUNG-PiB.

[57] GIJHARS (2017). Organic Agricultural Producers List-2016.

[58] Qiao Y., Martin F., Cook S., He X., Halberg N., Scott S., Pan X. (2018). Certified Organic Agriculture as an Alternative Livelihood Strategy for Small-Scale Farmers in China: A Case Study in Wanzai County, Jiangxi Province. Ecol. Econ..

[59] Zanoli R., Gambelli D., Vitulan S. (2007). Conceptual Framework on the Assessment of the Impact of Organic Agriculture on the Economies of Developing Countries.

[60] Cronbach L.J. (1988). Internal Consistency of Tests: Analyses Old and New. Psychometrika.

[61] Cronbach L.J. (1951). Coefficient Alpha and the Internal Structure of Tests. Psychometrika.

[62] Sijtsma K. (2009). On the Use, the Misuse, and the Very Limited Usefulness of Cronbach’s Alpha. Psychometrika.

[63] Singh A., Verma P. (2017). Factors Influencing Indian Consumers’ Actual Buying Behaviour towards Organic Food Products. J. Clean. Prod..

[64] GUS Average Monthly Gross Wage and Salary in National Economy in 2016 2017, 1. https://stat.gov.pl/en/latest-statistical-news/communications-and-announcements/list-of-communiques-and-announcements/average-monthly-gross-wage-and-salary-in-national-economy-in-2016,283,4.html.

[65] GUS (2017). Household Budget Survey in 2016.

[66] NBP (2016). Table A of Average Odds-Archive. https://www.nbp.pl/transfer.aspx?c=/ascx/ListABCH.ascx&Typ=a&p=rok;mies&navid=archa.

[67] Ostermann O.P. (1998). The Need for Management of Nature Conservation Sites Designated under Natura 2000. J. Appl. Ecol..

[68] Lomba A., Alves P., Jongman R.H.G., McCracken D.I. (2015). Reconciling Nature Conservation and Traditional Farming Practices: A Spatially Explicit Framework to Assess the Extent of High Nature Value Farmlands in the European Countryside. Ecol. Evol..

[69] Fischer-Hüftle P., Gellermann M. (2018). Landwirtschaft in Natura 2000-Gebieten. Nat. Recht.

[70] Gonthier D.J., Ennis K.K., Farinas S., Hsieh H.-Y., Iverson A.L., Batáry P., Rudolphi J., Tscharntke T., Cardinale B.J., Perfecto I. (2014). Biodiversity Conservation in Agriculture Requires a Multi-Scale Approach. Proc. R. Soc. B Biol. Sci..

[71] Tanentzap A.J., Lamb A., Walker S., Farmer A. (2015). Resolving Conflicts between Agriculture and the Natural Environment. PLoS Biol..

[72] Staniak M., Feledyn-Szewczyk B. (2016). Bioróżnorodność Obszarów Wiejskich-Znaczenie i Zagrożenia.

[73] Średnicka-Tober D., Obiedzińska A., Kazimierczak R., Rembiałkowska E. (2016). Environmental Impact of Organic vs. Conventional Agriculture—A Review. J. Res. Appl. Agric. Eng..

[74] Clark M., Tilman D. (2017). Comparative Analysis of Environmental Impacts of Agricultural Production Systems, Agricultural Input Efficiency, and Food Choice. Environ. Res. Lett..

[75] Habuda A., Biskup R., Pyter M., Rudnicki M., Trzewik J. (2014). Prawne Instrumenty Reglamentacji Działalności Gospodarczej Na Obszarach Chronionych. Działalność Gospodarcza na Obszarach Chronionych.

[76] Kemp D.R., Michalk D.L. (2007). Towards Sustainable Grassland and Livestock Management. J. Agric. Sci..

[77] Habel J.C., Dengler J., Janišová M., Török P., Wellstein C., Wiezik M. (2013). European Grassland Ecosystems: Threatened Hotspots of Biodiversity. Biodivers. Conserv..

[78] Hardelini J., Lankoskii J. (2018). Land Use and Ecosystem Services.

[79] Sienkiewicz–Paderewska D., Paderewski J., Suwara I., Kwasowski W. (2020). Fen Grassland Vegetation under Different Land Uses (Biebrza National Park, Poland). Glob. Ecol. Conserv..

[80] Kołodziejczak A., Rudnicki R. (2012). Environmentally-oriented common agricultural policy instruments and physical planning in agriculture. Acta Sci. Pol. Adm. Locorum.

[81] Hochkirch A., Schmitt T., Beninde J., Hiery M., Kinitz T., Kirschey J., Matenaar D., Rohde K., Stoefen A., Wagner N. (2013). Europe Needs a New Vision for a Natura 2020 Network. Conserv. Lett..

[82] IFOAM (2005). Principles of Organic Agriculture: Preamble.

[83] Heinrichs J., Kuhn T., Pahmeyer C., Britz W. (2021). Economic Effects of Plot Sizes and Farm-Plot Distances in Organic and Conventional Farming Systems: A Farm-Level Analysis for Germany. Agric. Syst..

[84] Lobley M., Butler A., Reed M. (2009). The Contribution of Organic Farming to Rural Development: An Exploration of the Socio-Economic Linkages of Organic and Non-Organic Farms in England. Land Use Policy.

[85] Tuomisto H.L., Hodge I.D., Riordan P., Macdonald D.W. (2012). Does Organic Farming Reduce Environmental Impacts?—A Meta-Analysis of European Research. J. Environ. Manag..

[86] Muri K., Tufte P.A., Coleman G., Moe R.O. (2020). Exploring Work-Related Characteristics as Predictors of Norwegian Sheep Farmers’ Affective Job Satisfaction. Sociol. Rural..

[87] Krot K., Glinska E. (2004). Uwarunkowania Subiektywnej Oceny Sytuacji Ekonomicznej Indywidualnych Gospodarstw Rolnych w Opinii Ich Właścicieli. Zesz. Nauk. SGGW-Ekon. Organ. Gospod. Żywn..

[88] Fiorelli C., Porcher J., Dedieu B., Darnhofer I., Grötzer M. (2010). Improving Farm Working Conditions: A Proposal to Characterise the Individual Relationship to Work. A Case Study Based on French Multi-Jobholder Sheep Farmers.

[89] Bouttes M., Bancarel A., Doumayzel S., Viguié S., Cristobal M.S., Martin G. (2020). Conversion to Organic Farming Increases Dairy Farmers’ Satisfaction Independently of the Strategies Implemented. Agron. Sustain. Dev..

[90] Tracy M. (1993). Food and Agriculture in a Market Economy: An Introduction to Theory, Practice and Policy.

[91] Besser T., Mann S. (2015). Which Farm Characteristics Influence Work Satisfaction? An Analysis of Two Agricultural Systems. Agric. Syst..

[92] Bórawski P., Bórawski M.B., Parzonko A., Wicki L., Rokicki T., Perkowska A., Dunn J.W. (2021). Development of Organic Milk Production in Poland on the Background of the EU. Agriculture.

[93] Komorowska D. (2015). Importance of Organig Farming in Poland. Ann. Pol. Assoc. Agric. Agribus. Econ. Ann. PAAAE.

[94] Cieślak I., Pawlewicz K., Pawlewicz A., Szuniewicz K. (2015). Impact of the Natura 2000 Network on Social-Economic Development of Rural Communes in Poland. Res. Rural Dev.-Int. Sci. Conf..

[95] Getzner M., Jungmeier M. (2002). Conservation Policy and the Regional Economy: The Regional Economic Impact of Natura 2000 Conservation Sites in Austria. J. Nat. Conserv..

[96] Gotkiewicz W., Sternik P. (2014). Environmental Awareness Among the Rural Population in Natura 2000 Areas, with Bartoszyce and Sorkwity Communes as an Example. Environ. Prot. Nat. Resour. Ochr. Śr. Zasobów Nat..

[97] Pawlewicz A., Cieślak I., Pawlewicz K., Szuniewicz K., Kusis J. (2015). Natura 2000 Sites and Socio-Economic Development of Rural Communes in Eastern Poland.

[98] Pawlewicz A., Pawlewicz K., Kościńska J. (2011). Funkcjonowanie Gospodarstw Rolnych Na Obszarach Natura 2000 w Opinii Rolników z Terenu Powiatu Olsztyńskiego. Pr. Nauk. Uniw. Ekon. We Wrocławiu.

